# HeptaTB Dx: a diagnostic model leveraging cuproptosis-ferroptosis crosstalk for distinguishing latent from active tuberculosis

**DOI:** 10.1128/spectrum.02995-25

**Published:** 2025-12-17

**Authors:** Linsheng Li, Peilong Wang, Zhiming Li, Guangliang Bai, Zhaoyang Ye, Ling Yang, Li Zhuang, Weiguo Sun, Wenping Gong

**Affiliations:** 1 Senior Department of Tuberculosis, Chinese PLA General Hospital 104607, Beijing, China; 2 Department of Respiratory & Critical Care Medicine, Hebei Chest Hospital 592467 , Shijiazhuang, Hebei, China; 3 Department of Clinical Laboratory, The Eighth Medical Center of PLA General Hospital 12509, Beijing, China; End TB Dx Consulting LLC, San Diego, California, USA

**Keywords:** cuproptosis, ferroptosis, latent tuberculosis infection, diagnostic model, biomarkers, immune infiltration

## Abstract

Distinguishing latent tuberculosis infection (LTBI) from active tuberculosis (ATB) remains challenging. The roles of cuproptosis-ferroptosis crosstalk in TB immunopathology and diagnostic potential are unexplored. Transcriptomic data from Gene Expression Omnibus data sets (GSE37250/GSE28623) were analyzed to identify cuproptosis-/ferroptosis-related differentially expressed genes. Bioinformatics (limma, weighted gene co-expression network analysis) and machine learning (LASSO, SVM-RFE) screened key biomarkers. A logistic regression model (HeptaTB Dx Model) was developed and validated in independent cohorts. Real-world validation included RNA-seq (*n* = 20) and reverse transcription quantitative polymerase chain reaction (RT-qPCR) cohorts (HC/LTBI/ATB, *n* = 111). Immune cell infiltration (ssGSEA/CIBERSORT) and consensus clustering elucidated mechanisms. We identified seven core biomarkers (MT1G, SCO2, CREB5, MGST1, PARP9, ATF3, and MUC1) regulating cuproptosis-ferroptosis interplay. HeptaTB Dx achieved exceptional performance: training, area under the curve (AUC) = 0.963 (sensitivity 0.928, specificity 0.897) and validation, AUC = 0.930 (sensitivity 0.920, specificity 0.870). Real-world RT-qPCR validation confirmed significant differential expression for 5/7 genes (CREB5, ATF3, MT1G, PARP9, and MGST1; *P* < 0.05) and model AUC = 0.778. Mechanistically, these genes formed a cooperative network linking immune regulation (ATF3/PARP9), ferroptosis suppression (MT1G/MGST1), barrier function (MUC1), and cuproptosis-metabolism (SCO2). CREB5 correlated with neutrophil infiltration (R = 0.83, *P* < 0.001), validating ferroptosis-immune crosstalk. LTBI subtypes exhibited divergent lipid metabolism-ferroptosis coupling and antiviral pathway enrichment. The HeptaTB Dx Model is the first diagnostic tool leveraging cuproptosis-ferroptosis crosstalk, offering high accuracy and mechanistic insights for LTBI management.

**IMPORTANCE:**

The differentiation between latent tuberculosis infection (LTBI) and active tuberculosis (TB) is a persistent challenge in global TB control, with current diagnostics failing to reliably distinguish these states or predict progression. This study introduces the HeptaTB Dx Model, the first diagnostic signature derived from the crosstalk between cuproptosis and ferroptosis—two metal-dependent regulated cell death pathways with emerging roles in *Mycobacterium tuberculosis* pathogenesis. By integrating seven key genes (MT1G, SCO2, CREB5, MGST1, PARP9, ATF3, and MUC1), the model achieves high diagnostic accuracy (area under the curve up to 0.963) and provides mechanistic insights into immune-metabolic dysregulation during TB infection. Validated in both public datasets and prospective clinical cohorts, HeptaTB Dx offers a scalable, transcriptome-based tool that outperforms existing single-pathway models and protein-based assays. This work not only advances TB diagnostics but also illuminates novel pathogenic mechanisms involving copper-iron interplay, with potential implications for therapeutic targeting.

## INTRODUCTION

Tuberculosis (TB), caused by *Mycobacterium tuberculosis* (MTB), remains a leading global health threat (1). An estimated 1.9 billion individuals—approximately one-quarter of the world’s population—carry latent tuberculosis infection (LTBI), of whom 5%–15% will progress to active tuberculosis (ATB) (2, 3). The World Health Organization reported 10.8 million incident cases and 1.25 million deaths in 2023 alone (4), underscoring the urgent need to identify and treat LTBI before progression occurs.

LTBI is defined as a persistent antigen-specific immune response without clinical or radiographic evidence of active disease (5, 6). Current diagnostics—tuberculin skin testing (TST) and interferon-gamma release assays (IGRAs)—lack gold-standard accuracy (7–10). TST, which relies on purified protein derivative, is vulnerable to false-negative results in immunocompromised hosts and to false-positive results through cross-reactivity with environmental non-tuberculous mycobacteria or prior Bacille Calmette–Guérin vaccination (9, 11, 12). Although next-generation kits have been commercialized, none reliably distinguish ATB from LTBI or predict which infected individuals will progress (6, 13). Consequently, robust biomarkers that discriminate LTBI from ATB are urgently required.

The molecular events that govern the transition from LTBI to ATB remain incompletely understood, but host–pathogen studies indicate substantial divergence at both transcriptomic and proteomic levels (1, 14, 15). MTB exploits multiple immune-evasion strategies—including manipulation of phagosome maturation, autophagy, and apoptosis—to persist within host macrophages (6). Emerging evidence underscores a potential synergistic crosstalk between ferroptosis and cuproptosis in the context of infectious diseases, particularly TB (16). These two metal-dependent cell death pathways, while distinct, share convergent points in cellular metabolism and stress responses (14, 17, 18). Copper and iron homeostasis are intrinsically linked, as exemplified by the role of copper-containing enzymes like ceruloplasmin in facilitating iron export, thereby influencing the labile iron pool available for ferroptosis (18, 19). Moreover, both pathways are potent inducers of mitochondrial dysfunction and oxidative stress (20, 21). Crucially, MTB has evolved sophisticated mechanisms to manipulate host metal metabolism for its survival (16). The pathogen not only sequesters iron to promote its replication but also upregulates copper efflux pumps (e.g., CtpV) to counteract host-mediated copper toxicity (16). This manipulation creates a unique immunometabolic landscape within infected macrophages, where dysregulated iron and copper levels may concurrently prime the cells for ferroptosis and cuproptosis death. Such synergistic dysregulation can dictate infection outcomes: while a balanced activation might aid in bacterial clearance, an excessive or dysregulated cell death can lead to tissue necrosis, inflammation, and bacterial dissemination, as seen in ATB (22). Therefore, deciphering the interplay between these pathways offers a novel, integrated perspective on TB pathogenesis, moving beyond single-pathway analyses to uncover a coordinated network of host cell death mechanisms that MTB exploits. This understanding provides a compelling rationale for constructing a diagnostic model that captures the essence of this crosstalk to better distinguish the tightly regulated state of LTBI from the dysregulated state of ATB.

Despite these insights, the diagnostic potential of the crosstalk between cuproptosis and ferroptosis remains unexplored. We therefore hypothesized that an integrated biomarker panel capturing this interplay could not only achieve superior diagnostic accuracy in distinguishing LTBI from ATB but also illuminate the critical molecular switch governed by metal ion homeostasis that underlies LTBI progression. Here, we interrogated publicly available transcriptomic data sets (Gene Expression Omnibus, GEO) from individuals with LTBI or ATB to identify differentially expressed ferroptosis- and cuproptosis-related genes (FRGs/CRGs). Using advanced machine-learning algorithms, we selected a minimal gene signature and constructed the HeptaTB Dx Model for LTBI/ATB discrimination. Immune-infiltration analyses revealed infection-associated microenvironmental shifts, and unsupervised clustering uncovered LTBI subgroups with distinct molecular profiles. Our findings nominate a novel dual-pathway biomarker set and offer mechanistic insight into the transition from latent to ATB.

## MATERIALS AND METHODS

### Study design and ethical review

Discovery of FRGs/CRGs biomarkers in this study utilized the GEO data set, while real-world cohort validation involved a prospective cohort study conducted at the 8th Medical Center of PLA General Hospital from October 2023 to February 2025. The study enrolled three groups: healthy controls (HCs), individuals with LTBI, and patients with ATB. Ethical approval was obtained from the Ethics Committee of the 8th Medical Center of PLA General Hospital (approval number: 30920230825701232), and all participants provided written informed consent, ensuring compliance with the Declaration of Helsinki.

### Data set selection and inclusion/exclusion criteria

#### Inclusion/exclusion criteria for the prospective cohort

HCs inclusion: (i) No history of TB exposure; (ii) negative ELISpot result; (iii) absence of TB symptoms and normal chest X-ray; and (iv) HIV-negative.

HCs exclusion: (i) Travel/residence history in high-TB-risk regions; (ii) age <12 years; (iii) history of TB or evidence of old TB lesions on chest imaging; (iv) contraindication/inability to undergo CFP-10/ESAT-6 (CE) antigen testing; and (v) HIV-positive.

LTBI inclusion: (i) Documented close contact with a smear-positive TB patient; (ii) absence of TB symptoms; (iii) normal chest X-ray; (iv) positive IGRA result; (v) HIV-negative; and (vi) age ≥12 years.

LTBI exclusion: (i) Diagnosis of ATB; (ii) pregnancy or lactation; (iii) HIV-positive; (iv) anti-TB treatment for ≥1 month; and (v) age <12 years.

ATB inclusion: Diagnosis of pulmonary, tracheobronchial, or pleural TB according to the Chinese National Standard for Tuberculosis Diagnosis (WS288-2017) (23), confirmed by positive mycobacterial culture or pathology, and supported by epidemiology, clinical presentation, imaging, and differential diagnosis.

ATB exclusion: (i) Systemic corticosteroid use; (ii) conditions affecting immune function (e.g., HIV infection, post-transplantation status, autoimmune diseases); (iii) malnutrition; and (iv) age <12 years.

#### GEO data set selection criteria

Transcriptomic data for biomarker discovery were obtained from the GEO database. Data sets were identified using the keywords “tuberculosis” and “latent tuberculosis infection. FRGs and CRGs were curated from a previous study (24).

Inclusion criteria: (i) Samples from ATB patients or LTBI individuals; (ii) ≥6 samples per group; (iii) complete gene expression profiles; (iv) age >15 years; (v) no prior TB treatment; (vi) blood samples from individuals without HIV, autoimmune diseases, or malignancy; and (vii) genome-wide RNA expression profiling.

Exclusion criteria: (i) Blood samples from immunocompromised individuals (e.g., HIV-positive, autoimmune disease, malignancy); (ii) samples from pregnant or lactating women; and (iii) samples from individuals aged <15 years.

ATB diagnosis (GEO): Positive MTB culture from respiratory samples, or culture-negative with characteristic TB symptoms/imaging and confirmed by clinical assessment.

LTBI diagnosis (GEO): Documented exposure to a smear-positive TB patient, positive TST or IGRA, absence of ATB symptoms or imaging findings.

The GSE37250 and GSE28623 data sets were selected as the primary training and validation sets, respectively, for the following reasons: (i) Both data sets utilize whole blood samples, which are clinically relevant for diagnostic biomarker discovery; (ii) they contain a sufficient number of well-defined LTBI and ATB samples meeting our inclusion criteria; and (iii) they have been widely used and validated in previous TB transcriptomic studies, supporting their reliability. To address potential batch effects arising from different platforms (GPL10558 for GSE37250 and GPL4133 for GSE28623), we performed a harmonization procedure. Gene symbols were used as common identifiers across data sets. For model validation, the coefficients from the logistic regression model trained on GSE37250 were directly applied to the normalized expression data of the seven core genes in GSE28623, a strategy that minimizes the impact of inter-dataset technical variation on the classifier performance. While this approach does not eliminate all non-biological variance, it allows for a robust assessment of the model’s generalizability across independent cohorts.

### Identification of differentially expressed genes and functional enrichment

GEO2R was used for initial analysis of microarray data. Differential expression analysis between ATB and LTBI samples in the GSE37250 data set was performed using the limma R package. Differentially expressed genes (DEGs) were defined by an adjusted *P*-value (adj. *P*) ≤0.05 and |log2 fold change (log2FC)| ≥1. Gene Ontology (GO) and Kyoto Encyclopedia of Genes and Genomes (KEGG) pathway enrichment analyses of DEGs were conducted using ClusterProfiler, with significance set at adj. *P* ≤ 0.05. Results were visualized using ggplot2 (volcano plots, heatmaps).

### Weighted gene co-expression network analysis

Weighted gene co-expression network analysis (WGCNA) was performed using the WGCNA R package to identify gene modules associated with LTBI. A scale-free network was constructed by selecting an appropriate soft-thresholding power (β) using the pickSoftThreshold function. An adjacency matrix was transformed into a topological overlap matrix, and hierarchical clustering with dynamic tree cutting identified co-expression modules. Module eigengenes were calculated, and similar modules were merged. Modules significantly correlated with the LTBI phenotype (|correlation coefficient| > 0.4, *P* < 0.05) were selected for further analysis.

### Screening and validation of LTBI-associated cuproptosis/ferroptosis key genes

Candidate key genes were identified by intersecting DEGs, genes from LTBI-associated WGCNA modules, FRGs, and CRGs using an online platform (Hiplot). To refine the candidate list and prevent overfitting, two feature selection algorithms were employed: (i) LASSO regression: Implemented with the glmnet package using 10-fold cross-validation to determine the optimal penalty parameter (λ). (ii) Support vector machine-recursive feature elimination (SVM-RFE): Implemented using the e1071 and caret packages, selecting features minimizing cross-validation error.

Genes selected by both LASSO and SVM-RFE were defined as core DEGs. Expression levels of core DEGs were validated using limma and ggpubr (*P* < 0.05). Diagnostic performance was assessed by receiver operating characteristic (ROC) curve analysis using the pROC package, calculating the area under the curve (AUC). Models were built and validated in independent data sets.

### Immune cell infiltration analysis

Immune cell composition in LTBI vs. ATB (GSE37250 data set) was quantified using single-sample gene set enrichment analysis (ssGSEA), evaluating 29 immune cell types. Results were visualized with heatmaps and violin plots (vioplot package). Spearman correlation analysis (reshape2, ggExtra) assessed associations between core DEGs and significantly altered immune cells. To validate ssGSEA results, the CIBERSORT algorithm was applied for deconvolution-based immune cell estimation.

The immune infiltration results obtained from ssGSEA and CIBERSORT were compared to assess their consistency. While both methods estimate immune cell abundances, they are based on distinct computational principles (ssGSEA on gene set enrichment and CIBERSORT on deconvolution using a signature matrix). We focused on the concordance in identifying the direction of change (enrichment or depletion) for key immune cell populations between LTBI and ATB groups across both methods. Any notable discrepancies between the two methods were discussed in the context of their technical differences.

### Identification of cuproptosis/ferroptosis subtypes in LTBI

Unsupervised consensus clustering of LTBI samples based on CRG/FRG expression profiles was performed using the ConsensusClusterPlus R package (k-means algorithm, 50 iterations). The optimal cluster number (k) was determined by the cumulative distribution function and consensus matrix heatmap analysis. Principal component analysis (PCA) in GraphPad Prism (v10.5.0) visualized sample distribution to confirm clustering stability and biological relevance.

### Functional enrichment and gene set variation analysis

GO and KEGG enrichment analyses of DEGs between identified LTBI subtypes were performed using ClusterProfiler (adj. *P* < 0.05). Gene set variation analysis (GSVA) was applied using the GSVA package to evaluate pathway activity variations across LTBI subtypes.

#### Diagnostic nomogram construction and evaluation

A diagnostic nomogram incorporating the core DEGs was constructed using the rms R package. Calibration curves assessed agreement between predicted and observed probabilities. Decision curve analysis (DCA) evaluated the clinical utility of the nomogram across a range of threshold probabilities.

### Validation of core gene expression in prospective cohorts

#### Participants

It is important to clarify the role of the HCs group in this study. The primary objective of our model is to discriminate between LTBI and ATB. Therefore, the HCs group was not used in the discovery, training, or primary validation of the HeptaTB Dx Model. The HC samples were included exclusively in the final reverse transcription quantitative polymerase chain reaction (RT-qPCR) validation phase to provide a biological reference point and to confirm that the expression trends of the core genes in LTBI and ATB differ from a non-infected state, thereby strengthening the clinical context of our findings.

Two sub-cohorts were enrolled: (i) RNA-seq validation: LTBI (*n* = 10) and ATB (*n* = 10); (ii) RT-qPCR validation: HCs (*n* = 37), LTBI (*n* = 37) and ATB (*n* = 37).

#### ELISpot assay

Peripheral blood (4 mL) was collected in heparin tubes. Peripheral blood mononuclear cells were isolated by Ficoll-Paque density gradient within 4 h, plated (2.5 × 10⁵ cells/well), and stimulated overnight with ESAT-6/CFP-10 peptide pools. IFN-γ-secreting cells were enumerated with MABTECH ELISPOT kits.

#### RNA extraction and quantification

Total RNA was extracted from 5 mL whole blood using the Simgen kit, treated with DNase I, and quantified on an IMPLEN spectrophotometer (A₂₆₀/₂₈₀ ≥1.8).

#### Reverse transcription and RT-qPCR

cDNA was synthesized with FastKing RT SuperMix. Quantitative PCR was performed on a QuantStudio 6 Flex using FastReal SYBR Green. Relative expression was calculated by the 2⁻ΔΔCt method with GAPDH as endogenous control. All reactions were run in triplicate.

### Statistical analysis

Data were analyzed using GraphPad Prism v10.5.0 and SPSS v27.0. Continuous variables were expressed as mean ± SD (normal distribution) or median (interquartile range, 25%–75%) (non-normal distribution). Count data as frequencies.

#### Group comparisons

For group comparisons, the following statistical tests were employed based on the data characteristics: an unpaired *t*-test for two groups with normal distribution, the Mann-Whitney U test for two groups with non-normal distribution, one-way ANOVA for three or more groups with normal distribution and equal variance, and the Kruskal–Wallis test for three or more groups with non-normal distribution or unequal variance. A *P*-value of less than 0.05 was considered statistically significant. .

#### Correlation analysis

Spearman’s rank correlation was used to assess the relationships between the expression levels of the seven core genes and the infiltration levels of immune cell subsets estimated by ssGSEA and CIBERSORT. Correlation coefficients (R) and *P*-values were calculated. A *P*-value < 0.05 was considered statistically significant. The correlation analyses were performed on the entire cohort (combining LTBI and ATB samples) to identify overarching relationships within the TB infection spectrum.

#### LTBI subtype analysis

PCA (GraphPad Prism) on core gene expression and principal components (PCs) with eigenvalues > 1 (Monte Carlo simulation, 1,000 permutations, 95th percentile) were retained.

#### Diagnostic model construction

Binary logistic regression (SPSS) using core gene expression levels; regression coefficients served as variable weights. Model performance was evaluated by ROC curve analysis (AUC, 95% CI, sensitivity, specificity; *P* < 0.05).

## RESULTS

### Identification of DEGs and functional enrichment

**Fig 1:**
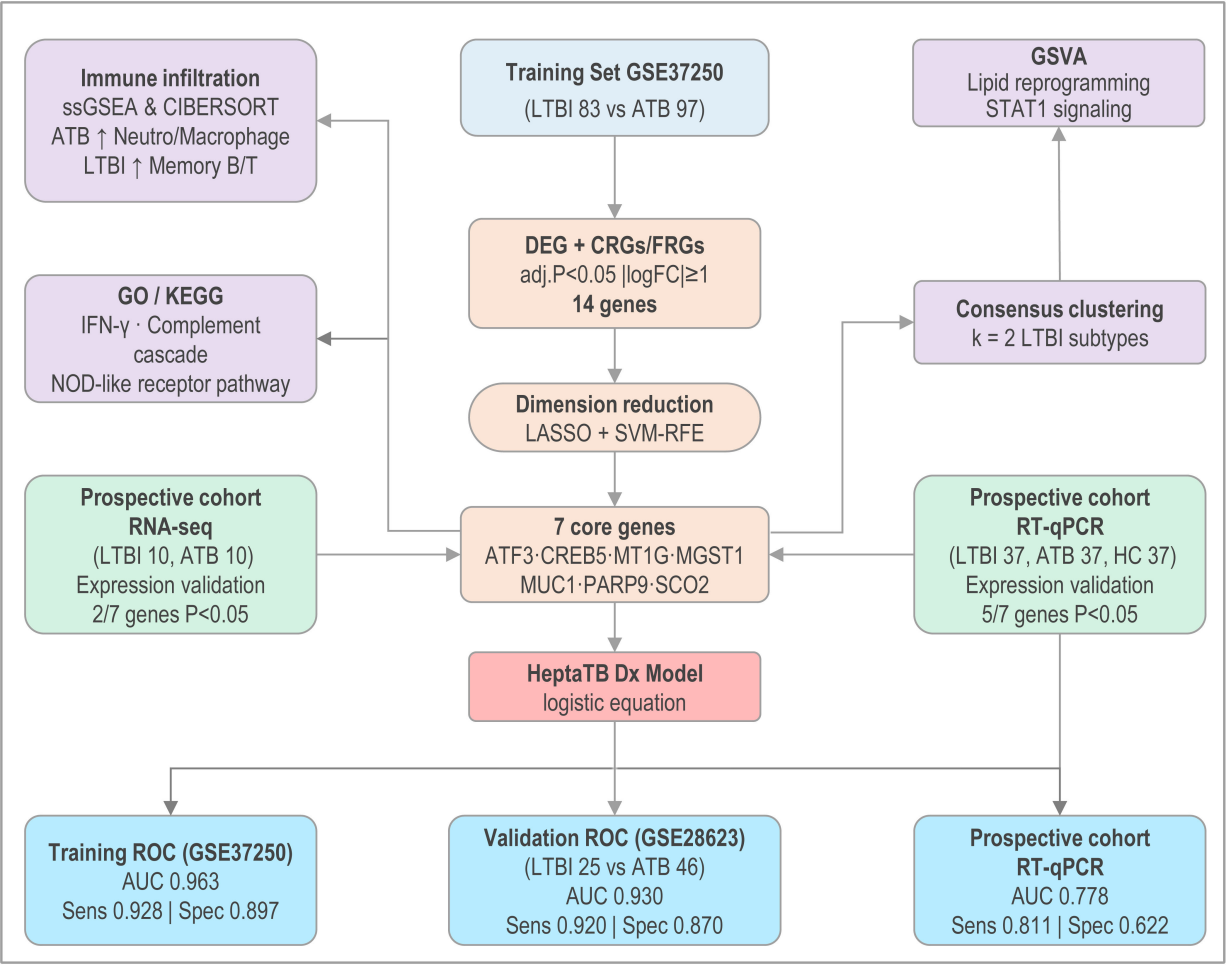
Schematic overview of the study design. The figure illustrates the workflow of the study, including the identification of DEGs related to cuproptosis and ferroptosis from the training set (GSE37250), validation in an independent cohort (GSE28623), and real-world validation using RNA-seq and RT-qPCR cohorts. The process involves bioinformatics analysis (limma, WGCNA), machine learning (LASSO, SVM-RFE), and immune cell infiltration analysis (ssGSEA, CIBERSORT) to develop the HeptaTB Dx Model. DEGs, differentially expressed genes; WGCNA, weighted gene co-expression network analysis; SVM, support vector machine; LASSO, least absolute shrinkage and selection operator; ATB, active tuberculosis; LTBI, latent tuberculosis infection; HC, healthy controls; ssGSEA, single-sample gene set enrichment analysis; CIBERSORT, immune cell infiltration analysis.

**Fig 2:**
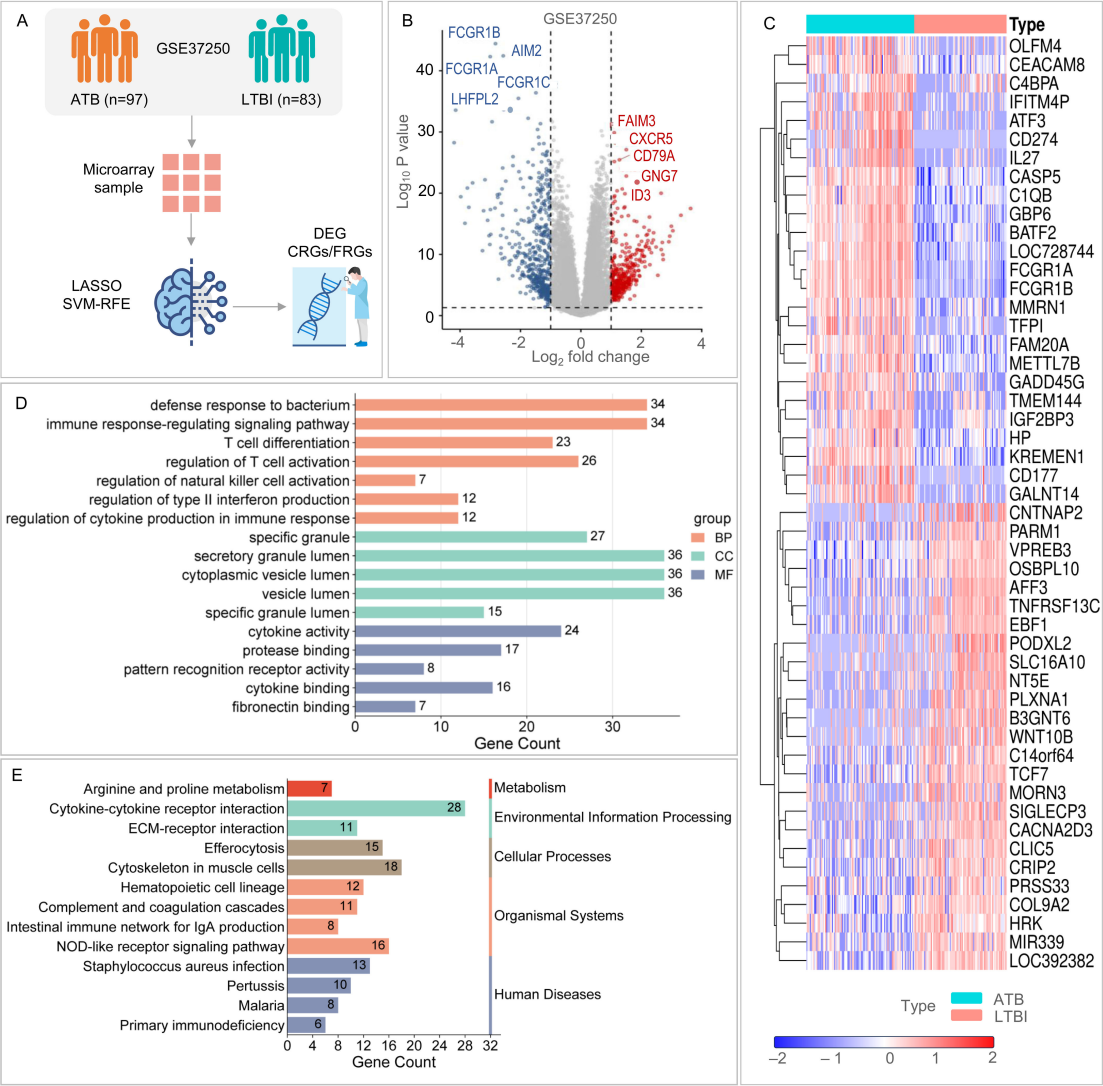
DEGs and GO/KEGG enrichment analysis in GSE37250. (**A**) Grouping and sample size in GSE37250. (**B**) Volcano plot of DEGs in GSE37250. (**C**) Heatmap of DEGs in GSE37250. (**D**) GO enrichment results for DEGs in GSE37250, with orange representing biological process, green representing cellular component, and blue representing molecular function. (**E**) KEGG pathway enrichment results. Adjusted *P* < 0.05 was considered significant. The x-axis represents the number of genes enriched for each term. The y-axis lists the GO-BP, GO-CC, GO-MF terms, and KEGG pathways (categorized into five classes). Bar lengths indicate the number of genes associated with each term. GO, Gene Ontology; KEGG, Kyoto Encyclopedia of Genes and Genomes.

A systematic search of GEO identified two independent data sets—GSE37250 (training) and GSE28623 (validation) (Fig. 1; Table 1). Limma analysis of GSE37250 revealed 925 DEGs (405 upregulated, 520 downregulated) between LTBI and ATB (Fig. 2A through C). ClusterProfiler enrichment indicated that these genes orchestrate bacterial defense, T-cell activation, type-II interferon signaling, granule secretion, and cytokine–cytokine receptor interaction (Fig. 2D and E).

**TABLE 1:** Summary of data set details

Data type	GEO series	Platform	Samples	Year	Tissue
Training set	GSE37250	GPL10558	LTBI: ATB = 83:97	2013	Blood
Validation set	GSE28623	GPL4133	LTBI: ATB = 25:46	2011	Blood

### WGCNA identifies key LTBI-associated modules

**Fig 3:**
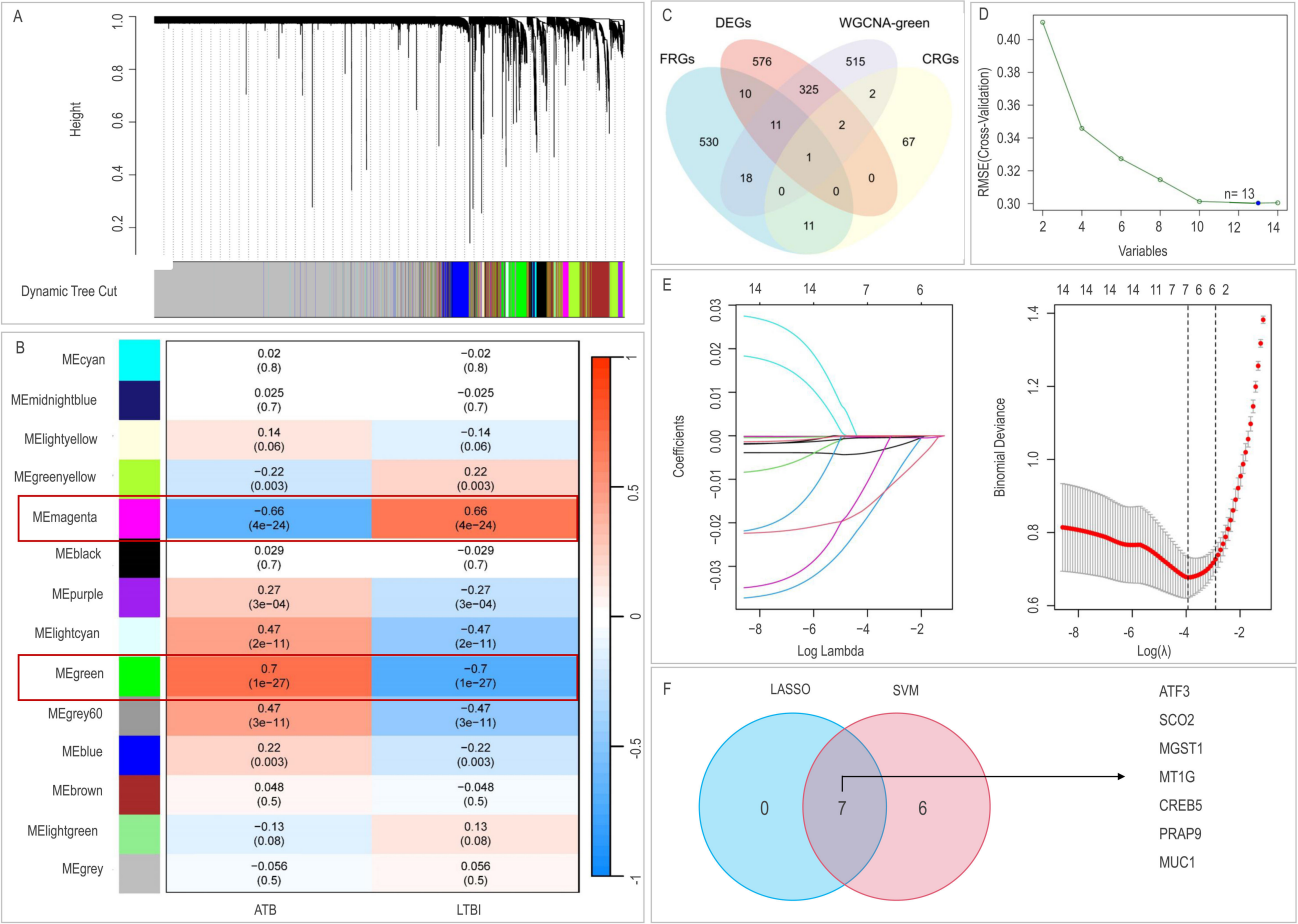
Identification of key LTBI modules in GSE37250 using WGCNA and machine learning for feature gene selection. (**A**) Gene clustering tree with modules color-coded. (**B**) Identification of key LTBI modules in GSE37250 using WGCNA. (**C**) Venn diagram of DEGs, WGCNA modules, cuproptosis, and ferroptosis genes. (**D**) Feature gene selection using SVM-RFE. (**E**) Feature gene identification using LASSO logistic regression with regularization parameter λ. (**F**) Venn diagram showing overlap of key feature genes selected by LASSO and SVM-RFE. WGCNA, weighted gene co-expression network analysis; SVM-RFE, support vector machine recursive feature elimination; CRGs, cuproptosis-related genes; FRGs, ferroptosis-related genes.

WGCNA partitioned genes into distinct co-expression modules (Fig. 3A). Module-trait relationship analysis identified the Magenta (positive correlation: r = 0.66, *P* = 4e-24) and Green (negative correlation: r = −0.7, *P* = 1e-27) modules as most significantly associated with LTBI status (Fig. 3B). Genes within these modules are likely central to LTBI pathophysiology.

### Identification of CRGs/FRGs

Intersection analysis of DEGs, genes from the highly correlated Green WGCNA module (874 genes), CRGs, and FRGs yielded 14 candidate genes (Fig. 3C): 11 FRGs (MUC1, MIR9-3, PARP9, CREB5, MGST1, LCN2, GALNT14, MAPK14, ATF3, MEG3, and SLC2A14), 2 CRGs (SCO2 and CD274), and MT1G (a common regulator). To refine the biomarker panel and mitigate overfitting, LASSO regression and SVM-RFE were employed. SVM-RFE identified 13 genes (*N* = 13, minimal classification error; Fig. 3D). LASSO regression selected seven genes (Fig. 3E). The intersection of both algorithms yielded seven core genes: MT1G, SCO2, MUC1, PARP9, CREB5, MGST1, and ATF3 (Fig. 3F).

### Construction and validation of the CRGs/FRGs-based LTBI diagnostic model

**Fig 4:**
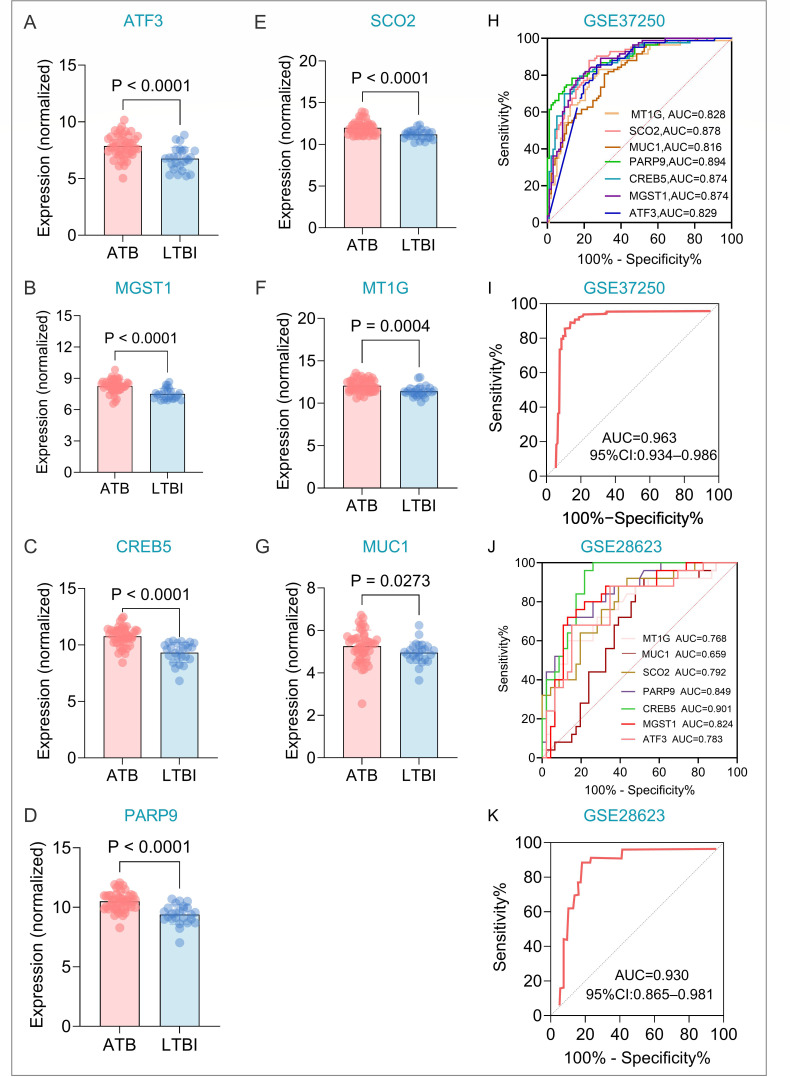
Expression validation and ROC analysis of core genes. (**A–G**) Expression of seven core genes in GSE28623. (**H–I**) Individual ROC curves for the seven core genes and the HeptaTB Dx Model in the training set GSE37250. (**J and K**) Individual ROC curves for the seven core genes and the HeptaTB Dx Model in the validation set GSE28623. AUC, area under curve; ROC, receiver operating characteristic curve.

Expression of all seven core genes was significantly different between ATB and LTBI in the validation set GSE28623 (SCO2, PARP9, CREB5, MGST1, ATF3: *P* < 0.0001; MT1G: *P* = 0.0004; MUC1: *P* = 0.0273; Fig. 4A through G). We constructed the logistic regression diagnostic model named the HeptaTB Dx Model:


$$P=\frac{1}{1+e^{-\left(31.875\;-\;0.795*MT1G\;+\;0.193*SCO2\;-\;1.102*MUC1\;-\;0.775*PARP9\;-\;0.624*CREB5\;-\;1.157*MGST1\;-\;0.284*ATF3\right)}}$$

where *P* represents the probability of LTBI, gene names denote expression levels, and *e* is the base of the natural logarithm.

In the training set, individual gene AUCs ranged from 0.816 to 0.894 (Fig. 4H). The combined HeptaTB Dx Model achieved excellent performance (AUC = 0.963, sensitivity = 0.928, specificity = 0.897, and accuracy = 0.911; Fig. 4I). This performance was robustly replicated in the validation set: individual AUCs 0.659–0.901 (Fig. 4J), combined model AUC = 0.930, sensitivity = 0.920, specificity = 0.870, accuracy = 0.887 (Fig. 4K).

### Immune infiltration landscape and molecular subtypes in LTBI

**Fig 5:**
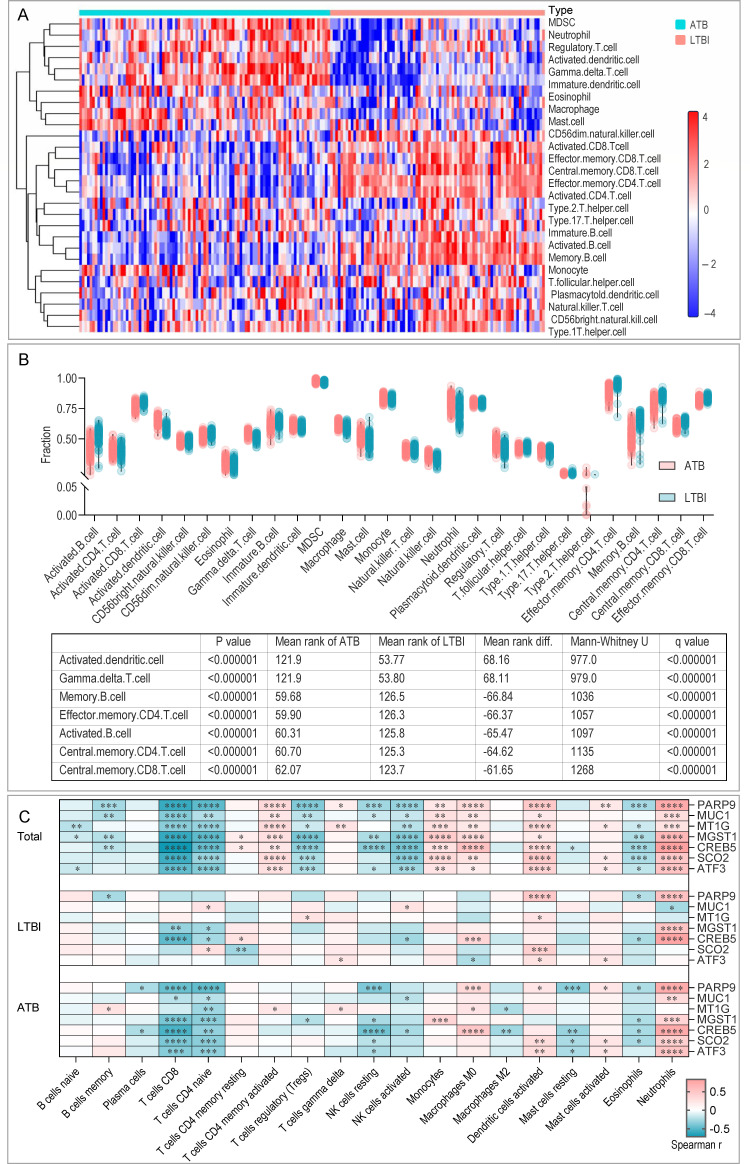
Immune cell infiltration analysis. (**A**) Heatmap of 28 immune cell types in ATB and LTBI. (**B**) Proportions of 28 immune cell types in ATB and LTBI. (**C**) Spearman correlation analysis between seven core genes and immune cells. Spearman correlation coefficients are color-coded, with red indicating strong positive correlation and blue indicating strong negative correlation. MDSC, myeloid-derived suppressor cells. *P < 0.05; **P < 0.01; ***P < 0.001; ****P < 0.0001.

To investigate the underlying immune mechanisms, we analyzed the immune cell infiltration patterns in LTBI and ATB. ssGSEA revealed distinct immune landscapes between the two states (Fig. 5A). ATB was characterized by a significant enrichment of innate immune cells, including activated dendritic cells and γδ T cells. In contrast, LTBI exhibited higher levels of adaptive immune cells, such as memory B cells and central memory CD4+and CD8+ T cells (Fig. 5B).

Spearman correlation analysis in the total cohort revealed extensive interactions between the seven core genes and immune cells (Fig. 5C). However, the number and strength of these significant correlations markedly decreased in the ATB subgroup and were further reduced in LTBI, indicating a simplification of the gene-immune interplay from active disease to latency. Notably, CREB5 exhibited the most robust correlations, maintaining strong positive associations with neutrophils and other innate cells, and negative associations with various T and NK cell subsets across all patient groups. This key relationship between CREB5 and neutrophils was validated by CIBERSORT analysis.

**Fig 6:**
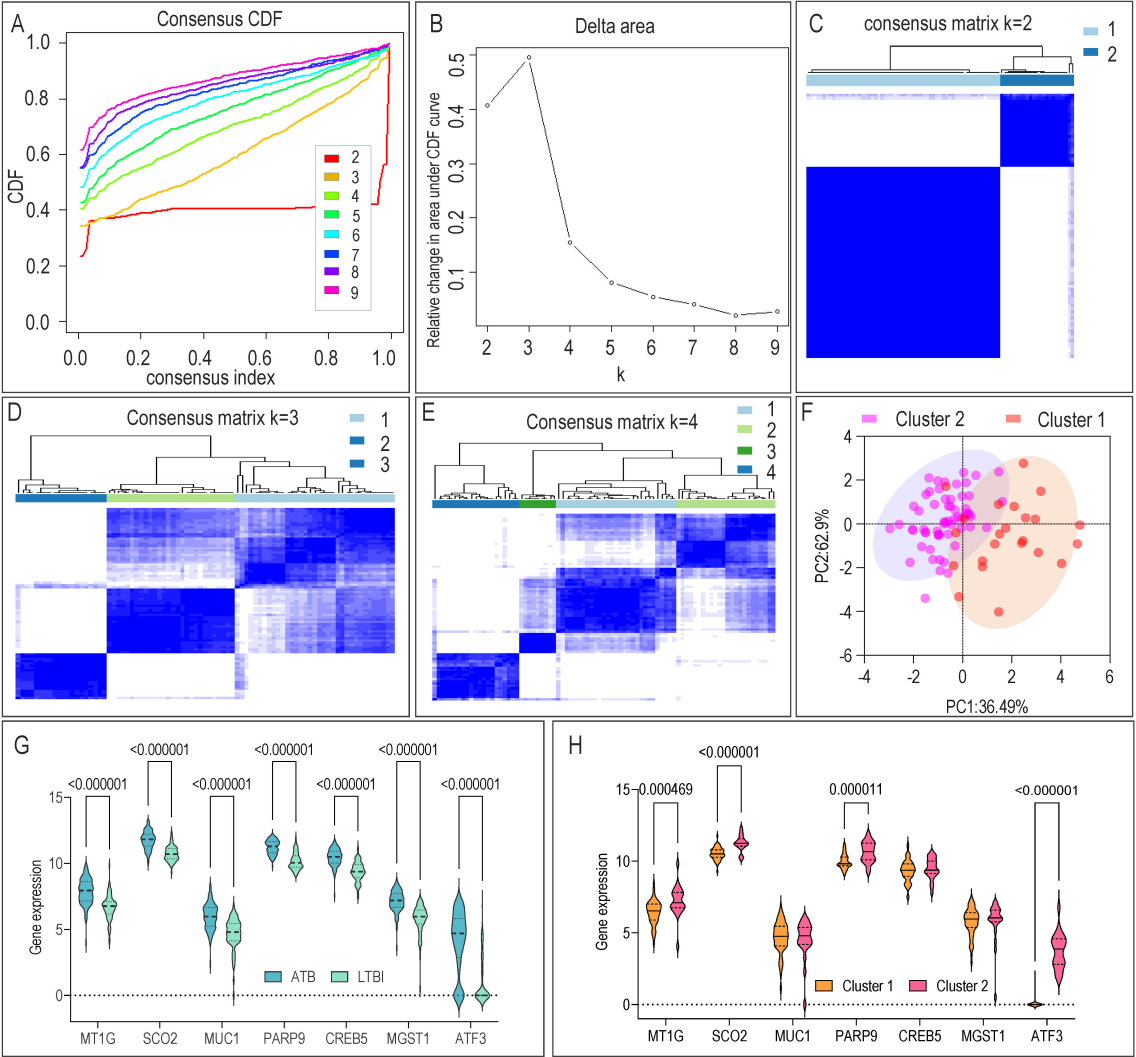
Identification of cuproptosis/ferroptosis-related molecular subtypes in LTBI and expression of seven genes across ATB, LTBI, and subtypes. (**A**) CDF curves showing consensus distribution from k = 2 to k = 9. (**B**) Area under the CDF curve for k = 2-9. The x-axis represents the number of clusters (k), and the y-axis represents the relative change in CDF area. (**C–E**) Consensus clustering matrices for k = 2–4. (**F**) PCA showing the distribution of two clusters. (**G**) Expression of seven core genes in ATB and LTBI. (**H**) Expression of seven core genes in cuproptosis/ferroptosis-related molecular subtypes. CDF, cumulative distribution function; PCA, principal component analysis.

Given the central role of CRGs/FRGs, we performed consensus clustering on the LTBI samples, which robustly identified two distinct molecular subtypes (Cluster 1 and Cluster 2; Fig. 6A through F). This analysis was performed exclusively on LTBI and ATB samples to delineate heterogeneity within the infection spectrum. As expected, all seven core genes were differentially expressed between ATB and the combined LTBI group (Fig. 6G). Importantly, within the LTBI cohort, four genes (MT1G, SCO2, PARP9, and ATF3) showed significant expression differences between the two subtypes (Fig. 6H), suggesting that these subtypes represent biologically distinct states of latent infection.

### Functional characteristics of LTBI subtypes and core genes

**Fig 7:**
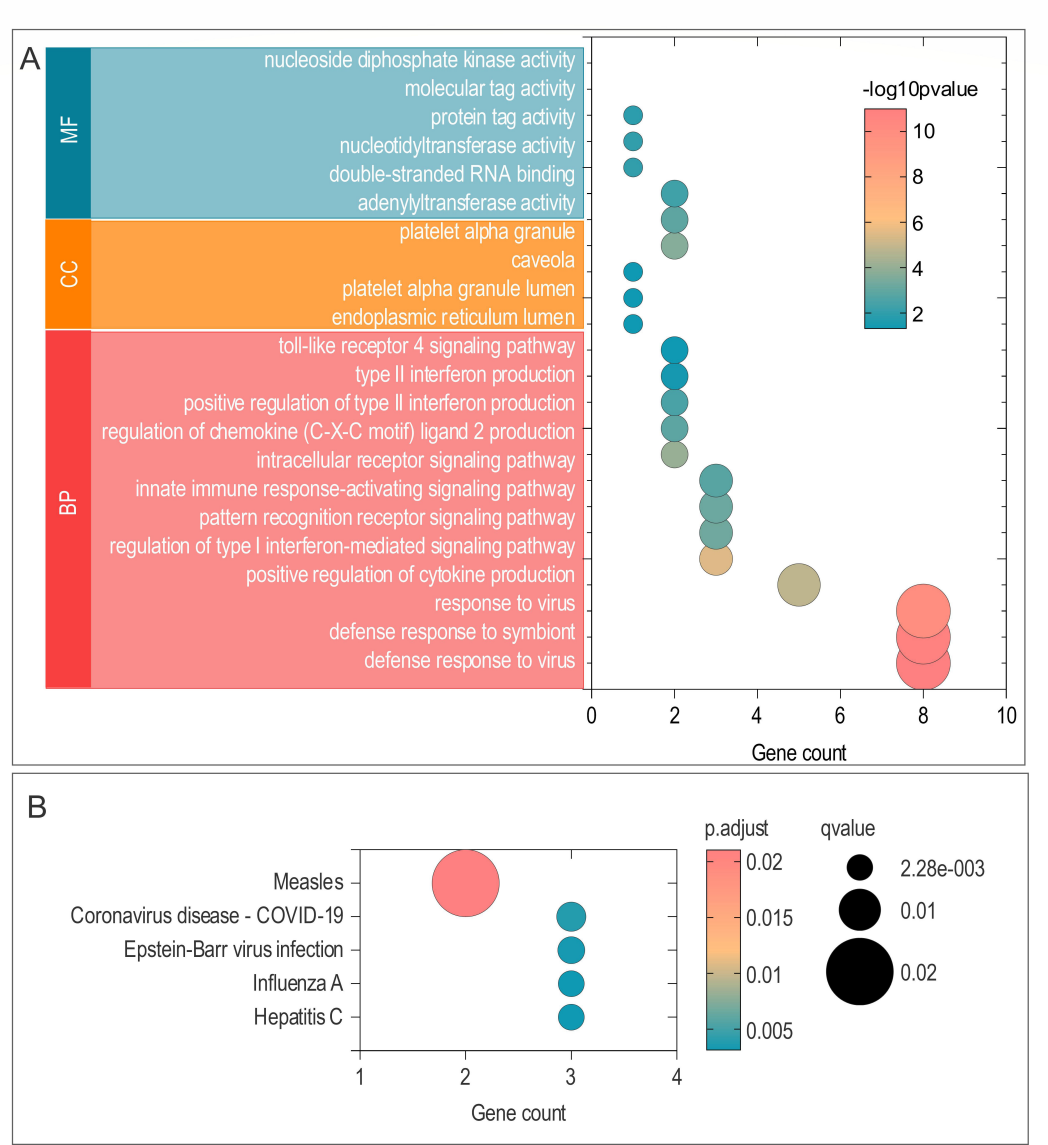
Enrichment analysis of differential genes between LTBI subtypes. (**A**) GO enrichment results for differential genes between clusters, with red representing biological process, yellow representing cellular component, and blue representing molecular function. (**B**) KEGG pathway enrichment results. Adjusted *P* < 0.05 was considered significant. The x-axis represents the number of genes enriched for each term. The y-axis lists the GO-BP, GO-CC, GO-MF terms, and KEGG pathways.

To functionally characterize the identified LTBI subtypes, we analyzed the DEGs between Cluster 1 and Cluster 2. GO enrichment analysis highlighted significant involvement in defense response to viruses, regulation of type I and II interferon signaling, and toll-like receptor pathways (Fig. 7A). KEGG analysis further showed enrichment for viral infection-related pathways, including Influenza and COVID-19 (Fig. 7B), indicating a heightened antiviral transcriptional state in one subtype.

**Fig 8:**
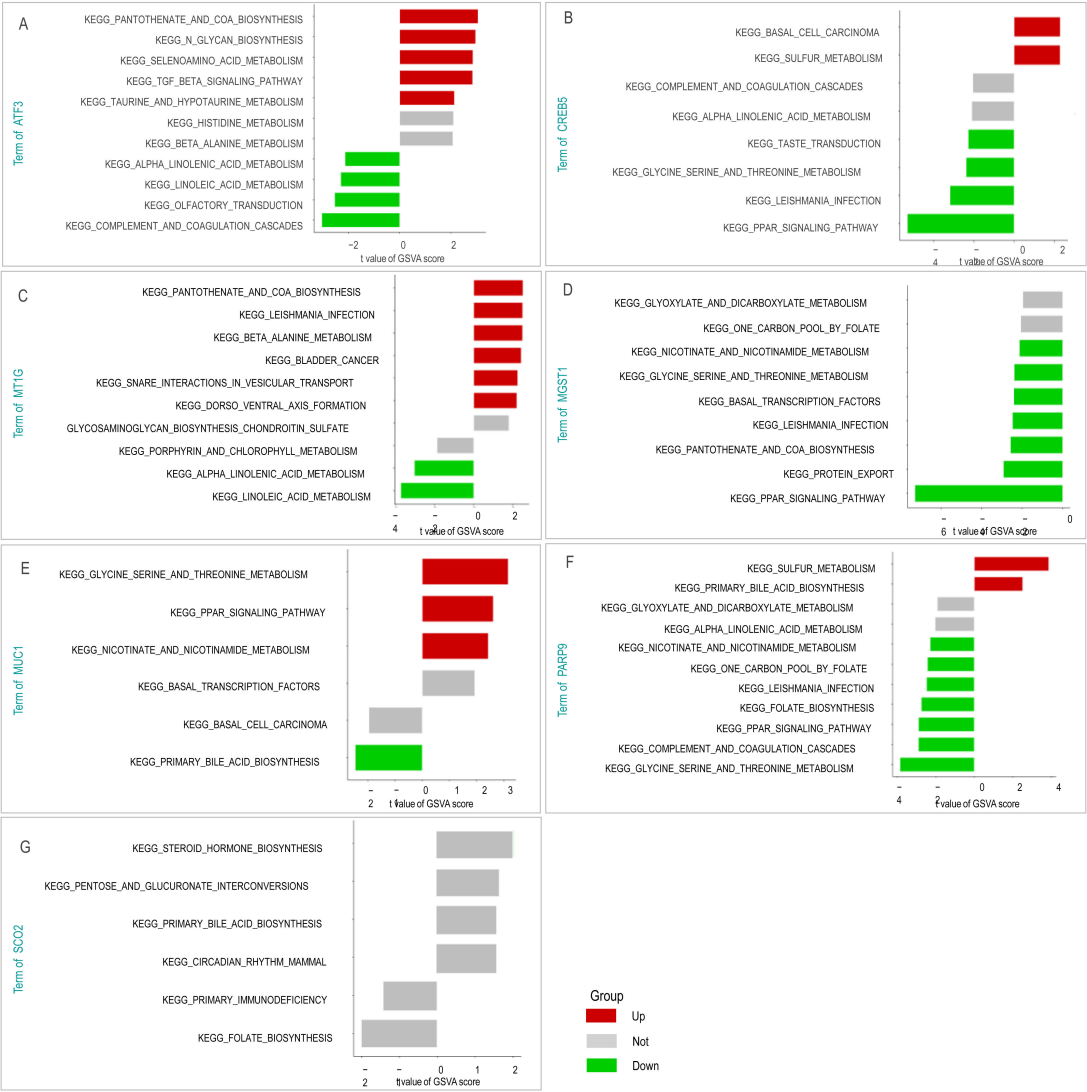
GSVA analysis of core genes ATF3 (**A**), CREB5 (**B**), MT1G (**C**), MGST1 (**D**), MUC1 (**E**), PARP9 (**F**), and SCO2 (**G**). The figure shows differences between high and low expression levels (top to bottom) of each gene. GSVA scores on the x-axis are ranked in descending order to reflect pathway enrichment levels, with two K-S statistical distribution lines plotted. GSVA, gene set variation analysis.

GSVA was then employed to uncover the potential biological functions of the individual core genes. This analysis suggested that these genes form a cooperative network (Fig. 8): (i) ATF3 and PARP9 were associated with immune modulation pathways (e.g., TGF-β signaling, complement cascade); (ii) CREB5, MT1G, and MGST1 were linked to anti-infectious responses and lipid metabolism (e.g., leishmaniasis, PPAR signaling); and (iii) MUC1 was implicated in barrier function and bile acid metabolism.

These results provide mechanistic insights into how the seven core genes may collectively regulate the immune-metabolic balance in TB infection, with ATF3 and PARP9 fine-tuning immune responses, while CREB5, MT1G, and MGST1 may coordinate oxidative stress and ferroptosis suppression.

### Clinical translation and validation of the HeptaTB Dx Model

**Fig 9:**
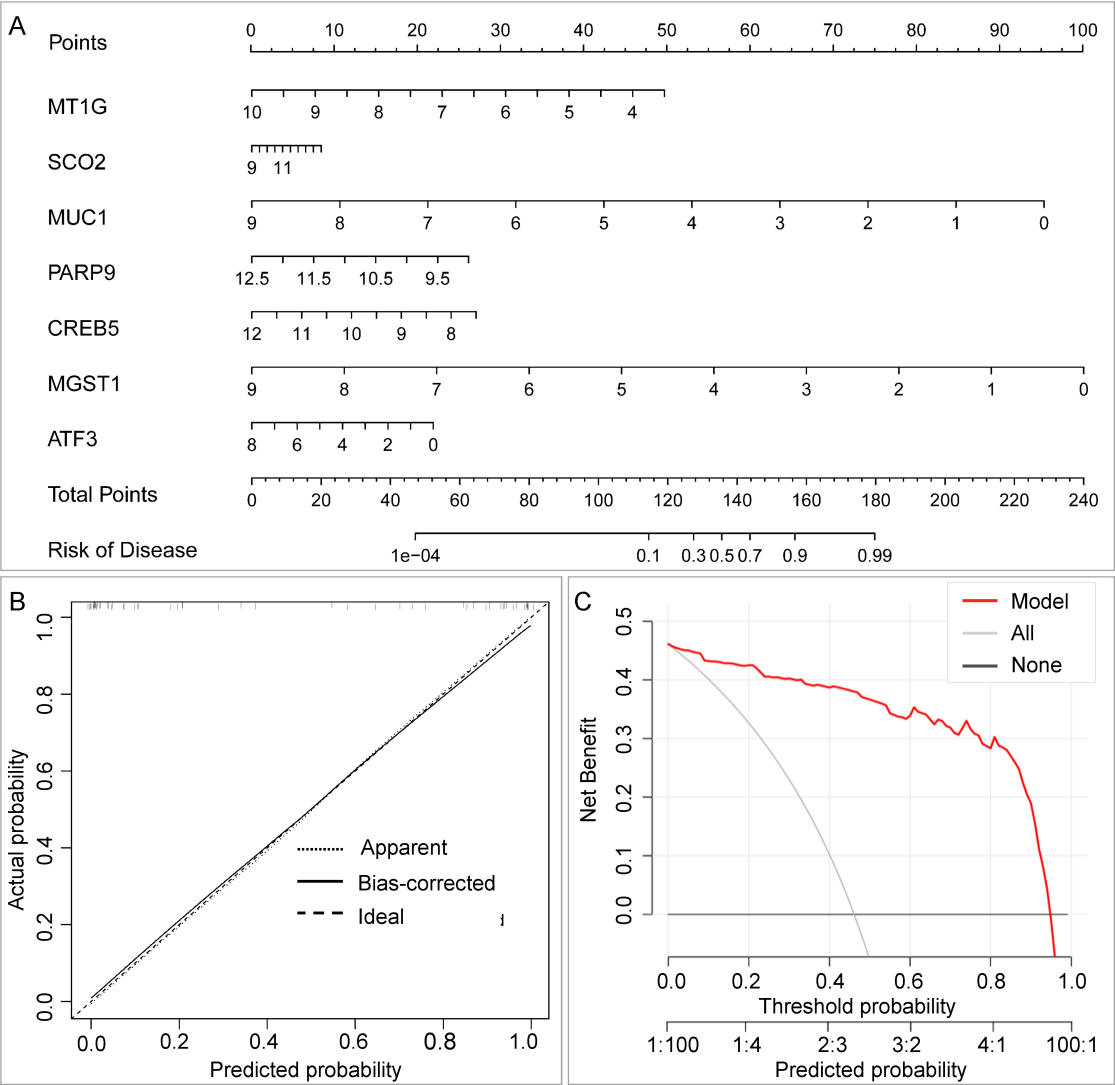
Nomogram of the HeptaTB Dx Model and validation for the seven core genes. (**A**) Nomogram of the HeptaTB Dx Model. (**B**) Calibration curve. (**C**) DCA.

To facilitate the clinical translation of our bioinformatics-derived signature, we constructed a diagnostic nomogram based on the HeptaTB Dx Model (Fig. 9A). This tool allows for the visual estimation of the individual probability of LTBI by summing points assigned according to the expression levels of the seven core genes. The nomogram demonstrated excellent calibration, with calibration curves showing strong agreement between the predicted probabilities and the actual observed outcomes (Fig. 9B). DCA further confirmed the clinical utility of the model, indicating a significant net benefit across a wide range of threshold probabilities (50%–99%), supporting its potential for informed clinical decision-making (Fig. 9C).

**Fig 10:**
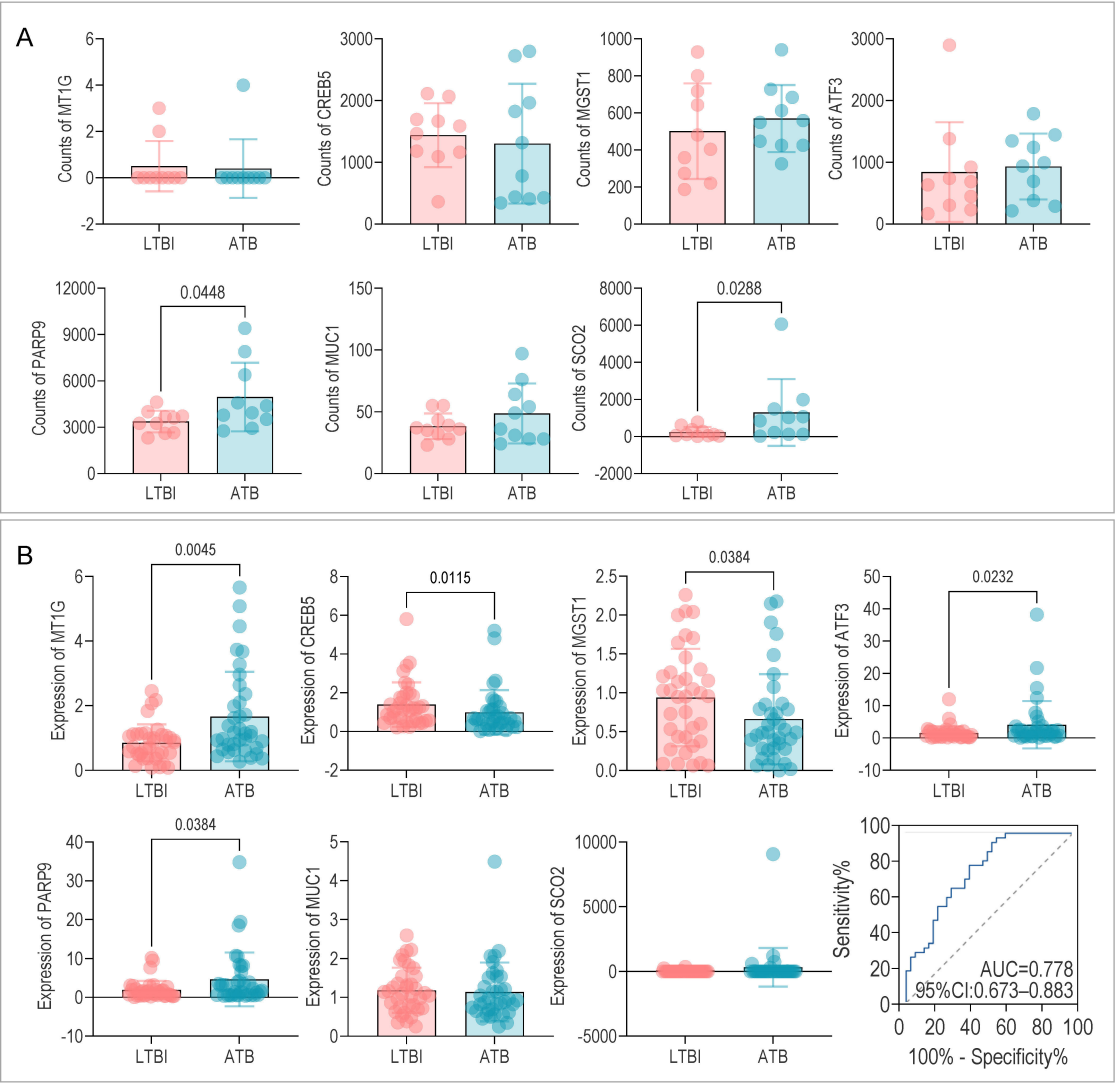
Expression analysis of seven DE-FRG/DE-CRG genes in ATB and LTBI using sequencing and RT-qPCR cohorts. (**A**) mRNA expression analysis of seven DE-FRG/DE-CRG genes in ATB and LTBI using sequencing data. (**B**) RT-qPCR validation of seven DE-FRG/DE-CRG genes in ATB and LTBI. DE-CRG, differential expression cuproptosis-related gene; DE-FRG, differential expression ferroptosis-related gene.

We next sought to validate the model and the expression of its constituent genes in two independent, prospective clinical cohorts. In the RNA-sequencing cohort (*n* = 20), the expression trends of the seven genes were consistent with our initial findings, with SCO2 and PARP9 achieving statistical significance (*P* < 0.05) despite the limited sample size (Fig. 10A). This validation was extended to a larger cohort using RT-qPCR (*n* = 111, including HC, LTBI, and ATB). In this analysis, five of the seven genes—CREB5, ATF3, PARP9 (downregulated in LTBI), and MT1G, MGST1 (upregulated in LTBI)—were confirmed to be significantly differentially expressed (*P* < 0.05) between LTBI and ATB, underscoring their robust roles in distinguishing infection states. While SCO2 and MUC1 showed consistent directional trends, they did not reach statistical significance in this cohort, a finding that may be elucidated in future studies with larger sample sizes.

Most importantly, when the combined HeptaTB Dx Model was applied to the RT-qPCR data, it achieved an AUC of 0.778 (95% CI: 0.673–0.883) in distinguishing LTBI from ATB, with a sensitivity of 81.1%, specificity of 62.2%, and an overall accuracy of 71.6% (Fig. 10B). This successful translation and validation in a real-world clinical setting confirm the model’s robustness and its potential as a practical diagnostic tool.

## DISCUSSION

This study introduces the first diagnostic model for TB that leverages the emerging paradigm of crosstalk between ferroptosis and cuproptosis. While ferroptosis is implicated in MTB pathogenesis (19), the role of cuproptosis—and critically, its interaction with ferroptosis—remains a key knowledge gap in TB immunobiology (25). Our work is grounded in the concept that the host’s simultaneous restriction of iron and deployment of copper as antimicrobial weapons creates a unique cellular environment, where dysregulation of both metals can conspire to drive pathogenic cell death and tissue damage. The HeptaTB Dx Model, by integrating biomarkers from both pathways, directly captures this critical interplay. Integrating seven CRGs/FRGs (MT1G, SCO2, CREB5, MGST1, PARP9, ATF3, and MUC1), it achieves dual superiority: exceptional diagnostic accuracy (AUC 0.930–0.963) and actionable mechanistic insights into LTBI progression—addressing a critical limitation of existing models that lack biological grounding despite high sensitivity (26).

The HeptaTB Dx Model demonstrates superior performance and mechanistic depth compared to existing diagnostic signatures for LTBI (Table 2) (27–36). While previous models have leveraged single biological processes—such as T-cell metabolism, neutrophil extracellular traps, or pyroptosis—their diagnostic accuracy is constrained by their narrow focus (27–30). Even integrated models based on immunity or autophagy have not matched the validation performance of HeptaTB Dx (AUC = 0.930) (31–33). Crucially, the most recent ferroptosis-specific signature, while conceptually aligned, lacks validation in prospective cohorts and overlooks the critical dimension of cuproptosis crosstalk (33). In contrast, protein-based assays like the IGRA-neutrophil nomogram, though accurate, rely on costly platforms that limit scalability (34, 35). Our transcriptome-based HeptaTB Dx Model achieves comparable high performance with greater potential for accessibility. It should be noted that the RT-qPCR validation cohort, while prospectively enrolled, was from a single center and of limited size (*n* = 111), which may affect the generalizability of the exact performance metrics (e.g., specificity = 0.622). Future multi-center studies with larger cohorts are warranted to refine these estimates.

**TABLE 2:** Performance and feature comparison of the HeptaTB Dx Model with existing diagnostic methods for discriminating LTBI from ATB^*a*^

Diagnostic method/signature	Basis/biomarkers	Reported AUC (range)	Sample type	Key strengths	Key limitations
HeptaTB Dx Model (this study)	Transcriptomic; 7-gene signature (CRG/FRG crosstalk)	0.930–0.963 (validation)	Whole blood	High accuracy; dual-pathway mechanistic insights; cost-effective (qPCR); validated in prospective cohort.	Specificity in real-world cohorts requires optimization; transcriptomic stability.
IGRA-based nomogram (34)	Protein; IFN-γ response & neutrophils	0.914	Whole blood	High accuracy.	Relies on costly protein detection platforms (e.g., Luminex/CLIA); limited mechanistic insight.
IFN-γ/IP-10 signature (35)	Protein; cytokine combination	0.955	Plasma	High accuracy.	Requires multiplex protein assay; higher cost and complexity.
T-cell metabolism genes (27)	Transcriptomic; five metabolic genes	0.867–0.873	Whole blood	Focus on T-cell immunity.	Single-process focus; lower AUC than HeptaTB Dx.
NETs-related biomarkers (28)	Transcriptomic; NETs-related genes	0.865–0.980	Whole blood	Focus on the neutrophil role.	Single-process focus.
Pyroptosis-related genes (29)	Transcriptomic; pyroptosis genes	0.787–0.946	Whole blood	Focus on pyroptosis pathway.	Single-process focus; performance varies.
Autophagy-related clusters (33)	Transcriptomic; seven autophagy genes	0.888	Whole blood	Focus on autophagy process.	Single-process focus; lower AUC than HeptaTB Dx.
Ferroptosis-specific signature (36)	Transcriptomic; four ferroptosis genes	0.715–0.991	Whole blood	Conceptually aligned with cell death focus.	Lacks prospective validation; overlooks cuproptosis crosstalk; performance range wide.

^*a*^ ATB, active tuberculosis; AUC, area under the curve; CRG, cuproptosis-related gene; FRG, ferroptosis-related gene; IGRA, interferon-gamma release assay; LTBI, latent tuberculosis infection; NETs, neutrophil extracellular traps.

Beyond performance, the HeptaTB Dx Model distinguishes itself by capturing the state of a synergistic gene network, offering unprecedented mechanistic insights into LTBI pathogenesis. The diagnostic power of the signature stems from its ability to reflect the integrity of this interactive network, which appears to be orchestrated through several key interactions:

Immune signaling directly regulates metal response. The interplay between ATF3, CREB5, and PARP9 forms a central immune-metabolic axis. ATF3 is a known negative regulator of CREB5 (37), and both are implicated in modulating the MAPK/PI3K-AKT pathway. Concurrently, the PARP9/STAT1 axis drives a Th1-polarizing, IFN-γ-rich environment (38, 39), which can directly influence the expression of metal-binding proteins and redox regulators. This creates a feed-forward loop where immune activation modulates metal homeostasis.

This immune context fine-tunes ferroptosis susceptibility. The antioxidant and ferroptosis-suppressive functions of MT1G and MGST1 are likely coordinated by the aforementioned immune signals. The IFN-γ signaling potentiated by PARP9 can influence the expression of metallothioneins like MT1G (40). Simultaneously, the lipid peroxidation suppressed by MGST1 is a key process in the ATF3-modulated inflammatory response (41, 42). Thus, the immune state set by the ATF3-PARP9 axis directly calibrates the ferroptotic susceptibility governed by MT1G/MGST1.

Cellular metabolism and barrier function are interdependent. The barrier integrity and direct antimicrobial effects of MUC1 are metabolically supported by SCO2. MUC1’s mucin barrier and β-defensin secretion are energy-demanding processes. SCO2, by ensuring efficient cytochrome c oxidase assembly and mitochondrial ATP production via copper delivery, provides the necessary metabolic foundation for MUC1’s functions (43, 44). Under the conditions of copper overload, the same SCO2-p53 axis can trigger cell death, linking copper metabolism directly to cell fate decisions.

In essence, the model captures a self-reinforcing network. Immune signals (ATF3/PARP9) regulate metal-handling and antioxidant genes (MT1G/MGST1), the functions of which are, in turn, supported by the metabolic activity of a CRG (SCO2). This core network is complemented by the physically protective barrier maintained by MUC1. Disruption of this delicate balance, as reflected in the combined signature score, heralds the transition to active disease.

Furthermore, our functional analyses validate clinical relevance: IFN-γ/NOD pathway enrichment directly reflects PARP9/STAT1 activity (38) and ATF3-mediated inflammation control (41, 45); CREB5-neutrophil correlation (R = 0.83) aligns with its role in ferroptosis suppression (46), explaining infiltrate differences in ATB vs. LTBI (6, 47, 48); LTBI subtypes show divergent lipid metabolism-ferroptosis coupling and viral defense pathway enrichment (e.g., TLR4, IFN-I/II), suggesting MTB co-opts antiviral mechanisms for latency.

This study has limitations. First, the training cohort’s ethnic homogeneity may affect the model’s generalizability, necessitating validation in larger, multi-ethnic cohorts. Second, the correlations between genes and immune cells, while insightful, require experimental validation to establish causality. Third, the precise molecular interplay (crosstalk) between ferroptosis and cuproptosis within the metabolic network of TB infection needs deeper mechanistic exploration. Future work should employ single-cell sequencing to dissect the dynamic regulation of these core genes within specific immune subsets (e.g., memory T cells, alveolar macrophages) and in response to therapeutic interventions.

### Conclusions

In conclusion, we developed the HeptaTB Dx Model, a novel diagnostic tool integrating CRG and FRG (MT1G, SCO2, CREB5, MGST1, PARP9, ATF3, and MUC1), which effectively distinguishes LTBI from ATB with superior performance (AUC = 0.963 in training, 0.930 in validation). This model captures crosstalk between metal ion homeostasis, oxidative stress, and immune regulation, offering mechanistic insights into TB pathogenesis. Validated in real-world cohorts, it outperforms single-pathway or protein-based assays in scalability and cost-effectiveness. These findings support its potential as a clinical tool for LTBI screening and inform future therapeutic targeting of metal-dependent cell death pathways.

## Data Availability

All data generated or analyzed during this study are included in this published article.
